# Analysis and classification of 1H-NMR spectra by multifractal analysis

**DOI:** 10.1371/journal.pone.0286205

**Published:** 2023-06-08

**Authors:** Jongphil Kim, Hin Kyeol Woo, Dixon Vimalajeewa, Brani Vidakovic

**Affiliations:** 1 Department of Biostatistics and Bioinformatics, H. Lee Moffitt Cancer Center & Research Institute, Tampa, FL, United States of America; 2 Department of Oncologic Sciences, Morsani College of Medicine, University of South Florida, Tampa, FL, United States of America; 3 Snapchat Inc., New York, NY, United States of America; 4 Department of Statistics, Texas A&M University, College Station, TX, United States of America; TU Wien: Technische Universitat Wien, AUSTRIA

## Abstract

The objective of this research focuses on the development of a statistical methodology able to answer the question of whether variation in the intake of sulfur amino acids (SAA) affects the metabolic process. Traditional approaches, which evaluate specific biomarkers after a series of preprocessing procedures, have been criticized as not being fully informative, as well as inappropriate for translation of methodology. Rather than focusing on particular biomarkers, our proposed methodology involves the multifractal analysis that measures the inhomogeneity of regularity of the proton nuclear magnetic resonance (^1^H-NMR) spectrum by wavelet-based multifractal spectrum. With two different statistical models (*Model-I* and *Model-II*), three different geometric features of the multifractal spectrum of each ^1^H-NMR spectrum (spectral mode, left slope, and broadness) are employed to evaluate the effect of SAA and discriminate ^1^H-NMR spectra associated with different treatments. The investigated effects of SAA include group effect (high and low doses of SAA), depletion/repletion effect, and time over data effect. The ^1^H-NMR spectra analysis outcomes show that group effect is significant for both models. The hourly variation in time and depletion/repletion effects does not show noticeable differences for the three features in *Model-I*. However, these two effects are significant for the spectral mode feature in *Model-II*. The ^1^H-NMR spectra of the SAA low groups exhibit highly regular patterns with more variability than that of the SAA high groups for both models. Moreover, the discriminatory analysis conducted using the support vector machine and the principal components analysis shows that the ^1^H-NMR spectra of SAA high and low groups can be easily discriminatory for both models, while the spectra of depletion and repletion within these groups are discriminatory for *Model-I* and *Model-II*. Therefore, the study outcomes imply that the amount of SAA is important and that SAA intake affects mostly the hourly variation of the metabolic process and the difference between depletion and repletion each day. In conclusion, the proposed multifractal analysis of ^1^H-NMR spectra provides a novel tool to investigate metabolic processes.

## 1 Introduction

High resolution spectroscopic data of bio-samples is a rich source of information on the metabolic response to physiological variation or pathological events [1]. In principle, spectroscopic outputs can provide a more integrated view of the bio-samples, enabling the extraction of valuable information regarding chemical balance quickly to identify individuals at risk, discovering potential drug targets, and assisting in management. A number of rapid and minimally invasive spectroscopic methods are described in Debik et al. [2] and Petricoin et al. [3]. However, this information is often exceedingly complex [4].

Analysis of spectroscopic data of bio-samples is a promising tool for identifying and evaluating potential biomarkers. The analysis of proton nuclear magnetic resonance (^1^H-NMR) spectroscopy and mass spectroscopy are the two main approaches typically used for metabolic spectroscopy data analysis [5]. The traditional approach in analyzing spectroscopic data from these two approaches is performed after the spectrum is subjected to a series of preprocessing steps, including smoothing, peak alignment, and normalization [2]. Even if the preprocessing steps are completed without errors, the measured response of a single chemical may not be fully informative due to various complex interactions. Although such interactions carry valuable information about specific biomarkers of interest, the traditional statistical techniques such as partial least squares-discriminant analysis do not capture such complex interactions effectively. In addition, translation of the methodology is difficult. There is high demand for more advanced spectroscopy data analyzing techniques.

Rather than focusing on particular biomarkers, this study proposes an approach that utilizes the global measure of spectral irregularity, overcoming the issues associated with the traditional techniques. The spectral irregularities are assessed by behavior of Fourier- or wavelet-spectra of the spectral output and summarized by the Hurst exponent, a dimensionless parameter between 0 and 1 that has been used in a range of disciplines. For example, when using ^1^H-NMR spectroscopy of plasma from individuals on chemically defined diets, Jung et al. [6] found that metabolic homeostasis is highly correlated with the Hurst exponent of the corresponding spectra. Similarly, Park et al. [7] showed that the Hurst exponent can be used to discriminate control subjects from cases by analyzing ^1^H-NMR spectra of their blood plasma. In addition, the study in Vimalajeewa et al. [8] used the Hurst exponent of protein mass spectra for the diagnosis of heart diseases and ovarian cancer, respectively.

It has been demonstrated that many processes encountered in nature, engineering, science, economics, etc., cannot be characterized by a single irregularity pattern (e.g., Hurst exponent). Such processes are typically characterized by complex scaling behavior, and this complexity of scaling is usually reflected by the inhomogeneity of the irregularity patterns. Theoretical arguments and statistical analyses have shown that many high frequency bioresponses are representative of these complex processes. A special technique, called “multifractal analysis”, was originally introduced by Frisch [9] to analyze geophysical measurements. The essence of multifractal analysis is the assumption about the singularities exhibited in the process, and this assumption is formed in the concept of the local strength of singularity organized in nested fractal sets. Thus, the multifractal analysis is focused not only on the irregularity or self-similarity of the data set described by a single parameter, but rather on a measure of inhomogeneity of such a parameter.

The rigorous mathematical theory of the multifractal process has been studied by many researchers [10, 11]. Considerable attention has been devoted to the study of dynamics of the multifractal processes [12, 13]. However, not much work has been done in extracting the quantified multifractal characteristics for an effective classification of the measurements taken from different categories, and the references are sparse.

Informally speaking, the multifractal spectrum describes the “richness” of the process in terms of local strengths of singularities. The term spectrum connotes the spectral decomposition of the process into components characterized by their irregularity. In that respect, the multifractal spectrum could be regarded as the frequency distribution of the local strength of singularities. The estimation of the multifractal spectrum relies on the multifractal formalism, which has been developed using advanced mathematical tools. The multifractal formalism can also be implemented in wavelet domains [14]. The advantage of using the wavelet-based multifractal spectrum is (i) the availability of the fast algorithm of wavelet transform, (ii) automatic separation of trends and fluctuations, and (iii) demonstrated effectiveness in a wide range of applications [14, 15]. This can be seen in multifractal characterization of the NMR T_2_ spectra of different types of coal for in-depth analysis of coal structures in Sun et al. [16] and Liu et al. [17]. Also, Ivanov et al. [18] reported an analysis for nutritional assessment by using multifractal-based features of ^1^H-NMR signal from human plasma. In addition, Soares et al. [19] utilized multifractal properties of images to propose a method to investigate spatial complexity of breast tumors.

The remainder of the paper is organized as follows. Section 2.1 considers an introductory overview of wavelet-based multifractal spectrum, while quantitative characteristics of the multifractal spectrum are discussed in Section 2.2. Section 3.1 describes the experiment and collection of data. Section 3.2 provides discussion of the results of the analysis by multifractal method. Final comments and conclusions are provided in Section 4.

## 2 Scaling, wavelets and multifractal spectrum

### 2.1 Wavelet-based multifractal spectrum

The wavelet-based calculation of multifractal spectrum relies on the concepts of *partition function* and *Legendre transform*. The partition function, *T*(*q*) is defined as

$$\begin{array}{c} T\left(q\right)=\underset{j\to \infty }{\text{lim}}(-\frac{1}{j}{\text{log}}_{2}\;E{\left|d_{jk}\right|}^{q}), \end{array}$$ (1)

where *d*_*jk*_ is a normalized wavelet coefficient corresponding to the wavelet basis that is *L*_1_ normalized, with basis functions *ϕ*_*jk*_(*t*) = 2^*j*^*ϕ*(2^*j*^*t* − *k*) and *ψ*_*jk*_(*t*) = 2^*j*^*ψ*(2^*j*^*t* − *k*) (if $d_{jk}^{*}$ are standard wavelet coefficients, the normalized are obtained as $d_{jk}=2^{-\frac{j}{2}}d_{jk}^{*}$). The moment order denoted by *q* is a real number within a certain range covering the negative numbers as well. However, the interpretation of the negative moments is not clear.

Even though (1) is very informative, the singularity measure is not explicit. It has been proposed in Goncalves et al. [20] that the local singularity strength could be measured in terms of wavelet coefficients as:

$$\begin{array}{c} \alpha \left(t\right)=\underset{k2^{-j}\to t}{\text{lim}}(-\frac{1}{j}{\text{log}}_{2}\left|d_{jk}\right|) \end{array}$$ (2)

where *d*_*j*,*k*_ is the normalized wavelet coefficient at scale *j* and location *k*. The local singularity strength measure (2) converges to the local Hölder index process at time *t*. Small values of *α*(*t*) reflect the more irregular behavior at time *t*. Any inhomogeneous process has a collection of local singularity strength measures, and their distribution *f*(*α*) forms the multifractal spectrum. A direct way to obtain this spectrum is to use the counting technique,

$$\begin{array}{c} f\left(\alpha \right)=\underset{\epsilon \to 0}{\text{lim}}\#\{\alpha (t)\;:\;\alpha -\epsilon <\alpha (t)<\alpha +\epsilon ,\;-\infty <t<\infty \}. \end{array}$$ (3)

Although it is feasible to estimate the multifractal spectrum using (2) and (3), the method is not practical due to the difficulty of approximating the limit, and also the significant computational complexity. A useful tool to make estimation efficient is the Legendre transform. The Legendre transform of the partition function is defined as

$$\begin{array}{c} f_{L}\left(\alpha \right)=\underset{q}{\text{inf}}\left\{q\alpha -T\left(q\right)\right\}. \end{array}$$ (4)

It can be shown that *f*_*L*_(*α*) converges to the true multifractal spectrum using the theory of large deviations [21].

From the practical point of view, we need a good estimator of the partition function. If we rearrange (1), it becomes

$$\begin{array}{c} E|d_{jk}{|}^{q}∼2^{-jT(q)}\;\text{as}\;j\to \infty . \end{array}$$ (5)

On the other hand, it has been shown by Arneodo et al. [14] that the *q*th moment of the wavelet coefficients of the power law process satisfies the following equation:

$$\begin{array}{c} E|d_{jk}{|}^{q}=C_{q}2^{-jqH} \end{array}$$ (6)

where *H* is the so-called self-similarity exponent and *C*_*q*_ is a constant depending only on *q* and choice of wavelet, but not *j*. Comparing (5) and (6), one can easily connect the partition function estimation with the self-similarity exponent estimation problem. It has been a standard practice to use linear regression to identify the self-similarity exponent *H* since the values *E*|*d*_*j*,*k*_|^*q*^ can be easily estimated by moment-matching method. This makes the estimation of the partition function *T*(*q*) straightforward. Formally speaking,

$$\begin{array}{c} {\text{log}}_{2}\;\hat{S_{j}\left(q\right)}=-jT\left(q\right)+\varepsilon_{j}, \end{array}$$ (7)

where $\hat{S_{j}\left(q\right)}=\frac{1}{2^{j}}\sum_{k=1}^{N2^{-j}}{\left|d_{jk}\right|}^{q}$ is the empirical *q*^*th*^ moment of the wavelet coefficients (*N* is the length of the time series) and the error term *ε*_*j*_ is introduced from the moment matching method when replacing the true moments with the empirical ones. Simple ordinary least square (OLS) is approximate but the most convenient choice the estimating the partition function.

Once *T*(*q*) is estimated, the next step is to perform the Legendre transform. Since $\frac{\partial }{\partial q}\left(\alpha q-T\left(q\right)\right)=\alpha -T^{\prime }\left(q\right)$ and *T*′′(*q*)<0, the maximum value of *αq* − *T*(*q*) is achieved at *q* = *T*′^(−1)^(*α*). So performing the Legendre transform is divided into two steps: First, the numerical derivative of *T*(*q*) is obtained using the finite differences. Then, the value of Legendre spectrum at $\alpha =\hat{T^{\prime }\left(q\right)}$ is evaluated. We point out that the Legendre transform is not able to estimate the multifractal spectrum value at arbitrary singularity strength *α*. The set of the multifractal spectrum values is determined by set of *q* values. The more *q* values adopted, the finer the multifractal spectrum obtained will be, i.e., the resolution of the spectrum is determined by the “(order) sampling frequency” of the moments.

### 2.2 Multifractal spectral characteristics

**Fig 1:**
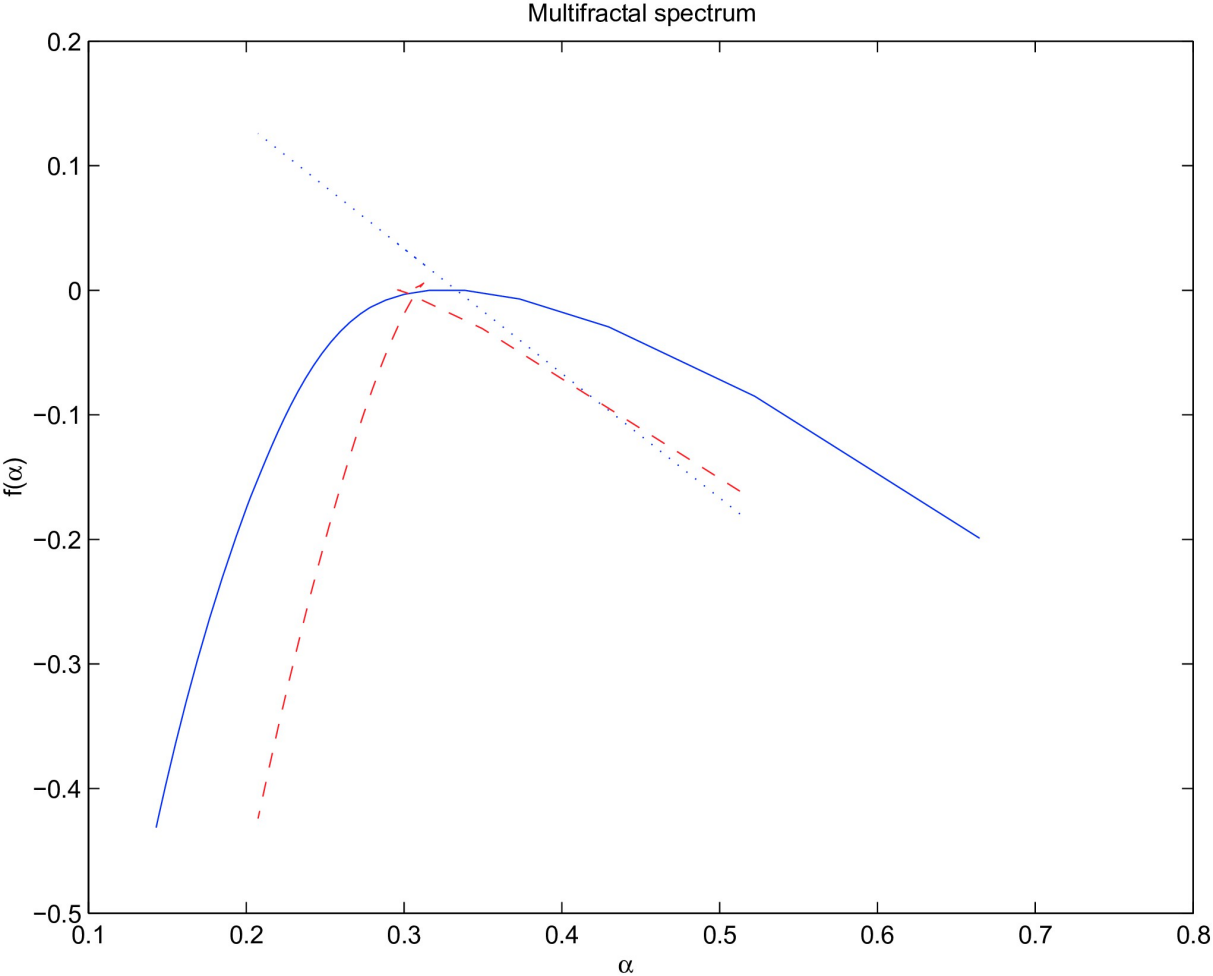
Multifractal spectra for monofractal process (dashed line) and turbulence signal (solid line). The dotted line indicates the theoretical slope of the spectrum for the monofractal process).

Rather than operating with multifractal spectra as functions (densities), we summarize them by a small number of meaningful descriptors. These descriptors have interpretation in terms of location of and definition from monofractality. Definition and analysis of multifractal spectra descriptors is one of contributions of this paper. Theoretically, the multifractal spectrum of fBm (a theoretical representative of a monofractal) consists of three geometric parts: the vertical line, the maximum point and the right slope [20]. The maximum point corresponds to the Hurst exponent and the vertical line and the right slope are thought to be inherent features, which distinguish fBm from the multifractal process. However, it is rare to obtain such a perfect spectrum in practice. Even for the well simulated fBm, due to errors in estimation (most of them due to the partition function estimation and derivative calculation as shown above), its spectrum may deviate from the theoretical form, as shown in Fig 1. Even with the lack of precise estimation of the spectrum, the extent of deviation from the vertical line could still be utilized in the discrimination between the monofractal and multifractal processes. For example, two type processes are presented in the multifractal spectra in Fig 1. One is the fBm and the other is the turbulence measurement, which is commonly believed to be a multifractal process. Both time series share the Hurst exponent 1/3 and have indistinguishable second-order properties. Compared to the turbulence signal, the fBm is much closer to the vertical line. This closeness may be quantified by the left slope of the spectra. Another important difference between these two spectra is the width (spread) of the spectra. It is obvious that the width of the fBm is much smaller than that of the turbulence signal indicating the richness of singularity indices in the later.

**Fig 2:**
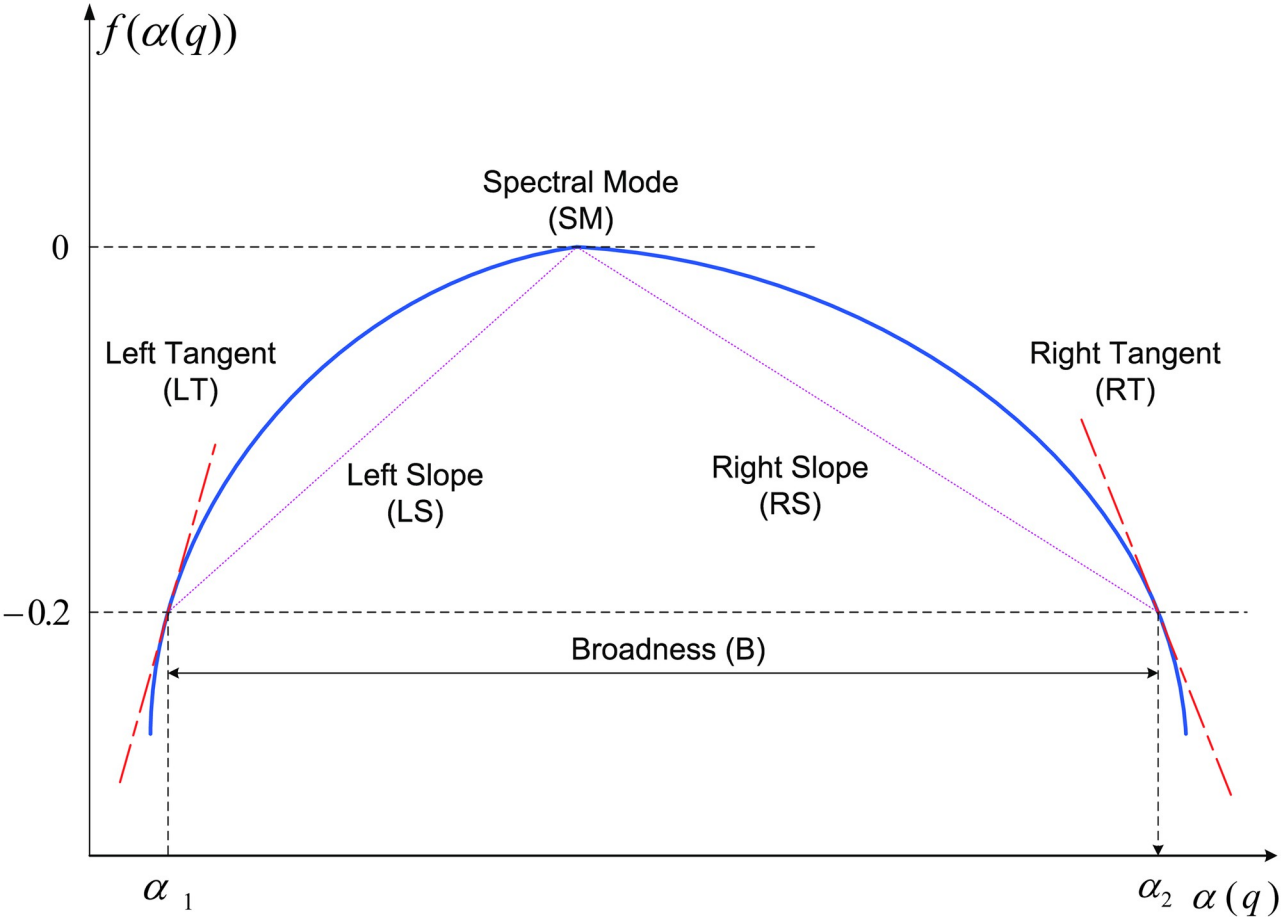
Illustration of geometric descriptors of multifractal spectra. Note that horizontal axis represents values of Hölder regularity index *α*(*q*), while vertical axis represents values proportional to the relative frequency of these indices, *f*(*α*(*q*)).

Despite the existence of the estimation error, the multifractal spectrum can be approximately described by 6 canonical descriptors without loss of the discriminant information. The descriptors are (1) Spectral Mode (Hurst exponent, *SM*), (2) left slope (*LS*), (3) left tangent (*LT*), (4) width (Broadness, *B*), (5) right slope (*RS*), and (6) right tangent (*RT*). A typical multifractal spectrum can be quantitatively described as shown in Fig 2. Understanding the *SM* and *LS* (or *LT*) is straightforward. *SM* represents the apex of spectrum or most common Hölder regularity index *α* found within the signal, and *LS* (or *LT*) represents the slope of the distribution produced by the collection of Hölder regularity index *α* with smaller values of the mode (*SM*). However, broadness (*B*) is more intricate descriptor of the multifractal spectrum. Broadness (*B*) is believed to be a more meaningful descriptor than right slope (*RS*) or right tangent (*RT*) because it is a compound measure representing the overall nature of the multifractal spectra, taking into account the overall variability among the Hölder regularity index *α*. In addition, broadness (*B*) partially accounts for right slope (*RS*) or right tangent (*RT*) in calculation, as the resultant value of *B* is based on the relative values of *RS* and *LS*. In this study, we choose the left slope (*LS*), Spectral Mode (*SM*) and the broadness (*B*) as the spectral characteristics because we believe that the width spread has more power to discriminate the multifractality.

Both slopes (or both tangents) can be obtained easily using the interpolation technique, but it is not straightforward to define broadness (*B*) automatically. The difficulties are related to two aspects—(i) difficulty to locate the start and end points of the width spread, and (ii) treatment of the discreteness of the spectrum. It is easy to see that the former is conceptually difficult, while the latter is computationally difficult. There are many ways to define the broadness (*B*). In this paper, we select the following definition [22].

**Definition** Suppose that *α*_1_ and *α*_2_ are two roots which satisfy the equation *f*(*α*) + 0.2 = 0 and *α*_1_ < *α*_2_. The broadness (*B*) of multifractal spectrum is defined as *B* = *α*_2_ − *α*_1_, where *f*(*α*) is the multifractal spectrum as a function of Hölder regularity indices *α*.

This definition with two tangents and two slopes is also graphically presented in Fig 2. The deviation from a monofractal could be assessed according to this broadness measure. It is worth noting the threshold value 0.2 used in this definition could be adjusted empirically in the practice analysis to ensure that this measure is well defined for all analyzed signals. The choice of threshold value is correlated back to the choice of *q* and the inherent data characteristics because these are the factors that affect the resolution of the spectrum.

As mentioned earlier, the discreteness may produce difficulties in computation. The problem is that it may be hard to find the exact roots of the equation *f*(*α*) + 0.2 = 0 among the discrete values of *α*’s. To get around this, we first find two closest points $\left(\alpha_{i}^{l},\;f\left(\alpha_{i}^{l}\right)\right)$ and $\left(\alpha_{i}^{u},\;f\left(\alpha_{i}^{u}\right)\right)$ for each *i* such that

$$\begin{array}{c} f\left(\alpha_{i}^{l}\right)<-0.2\;\;\text{and}\;\;f\left(\alpha_{i}^{u}\right)>-0.2\;i=1,2. \end{array}$$

The two solutions *α*_1_, *α*_2_ can be easily obtained by interpolation. Thus two slopes *LS*, *RS* and two tangents *LT*, *RT* can be easily obtained by

$$\begin{array}{ccc} LT & = & \left(f\left(\alpha_{1}^{u}\right)-f\left(\alpha_{1}^{l}\right)\right)/\left(\alpha_{1}^{u}-\alpha_{1}^{l}\right)\;\;\text{and}\;\\ RT & = & \left(f\left(\alpha_{2}^{u}\right)-f\left(\alpha_{2}^{l}\right)\right)/\left(\alpha_{2}^{u}-\alpha_{2}^{l}\right).\\ LS & = & 0.2/(SM-\alpha_{1})\;\;\text{and}\;\\ RS & = & -0.2/(\alpha_{2}-SM). \end{array}$$

## 3 Analysis and classification of ^1^H-NMR spectra by multifractal analysis

### 3.1 Data collection and experimental design

Motivated by the fact that a large number of biologic systems are functionally dependent upon sulfur amino acids (SAA) nutrition, researchers in the Clinical Biomarkers Laboratory at Emory University conducted metabolomics research to identify metabolic changes associated with SAA deficiency. This research is important for nutritional support because one of the SAA, cysteine, is routinely omitted due to its instability in nutritional support formulas. The ^1^H-NMR spectroscopy was selected because of its speed and simplicity in sample processing and its ability to obtain information on macronutrition that is quantitatively comparable over time. The ^1^H-NMR spectral data were provided from previously published studies by Jones et al. [23] and Park et al. [24] that were reviewed and approved by the Emory University Investigational Review Board.

**Fig 3:**
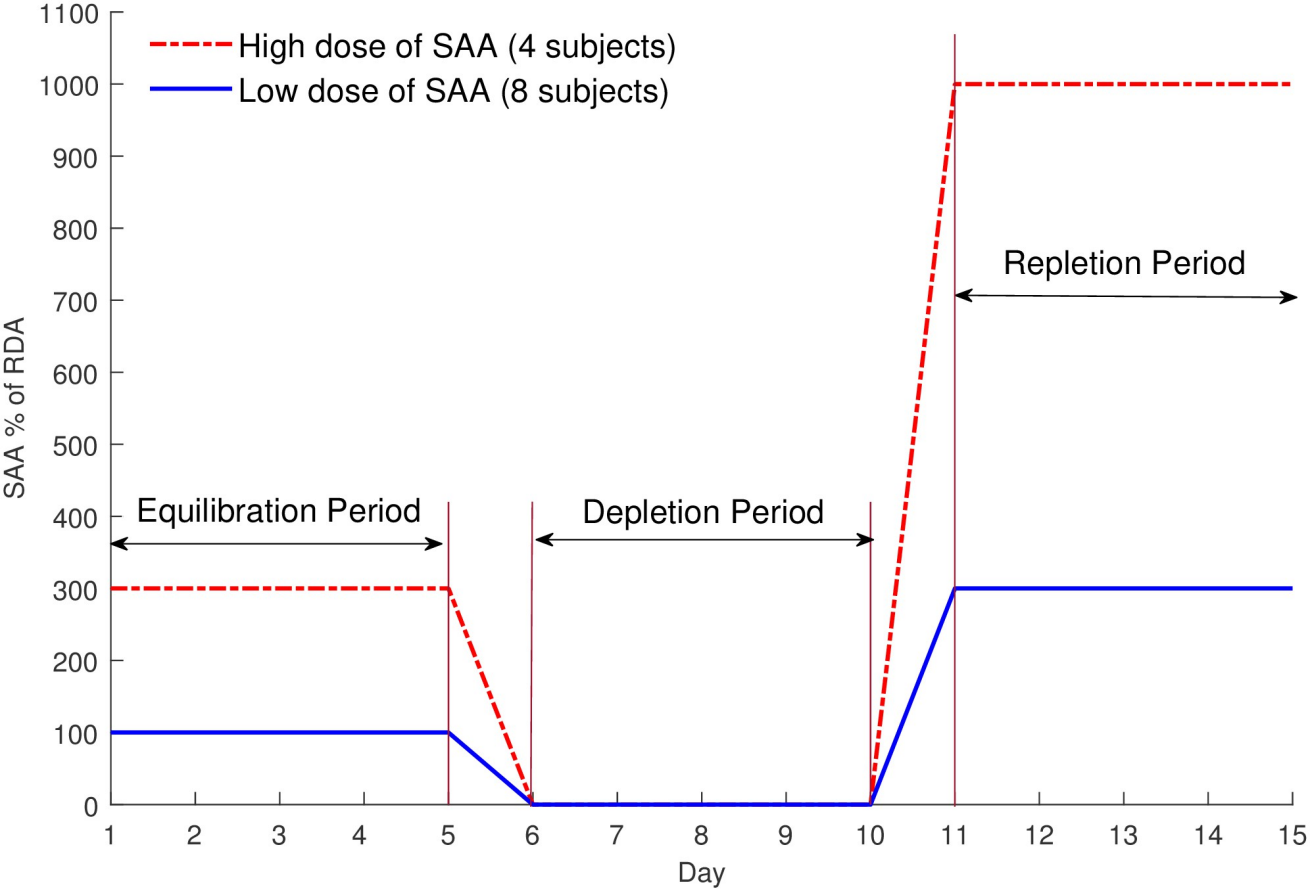
Experiment design: Data were collected through 3 phases; equilibration, depletion and repletion (decuple or treble recommended dietary allowance (RDA)) of SAA.

To determine whether the variation in the amount of SAA intake affects metabolic processes, researchers conducted the study with two study groups (high and low dose of SAA group) and with three phases as shown in Fig 3. The first phase was an equilibration period in which subjects received the treble recommended dietary allowance (RDA) for SAA (High group, 4 subjects) or 100% RDA for SAA (Low group, 8 subjects) for 5 days to equilibrate their diet. For the final 48 hours of the this period subjects were admitted as inpatients to the GCRC (General Clinical Research Center at Emory University) and the identical diet was continued. Following the equilibration period, subjects were placed on the 0% SAA diet for the 5-day depletion period, followed by the diet with either decuple RDA for SAA for High group or treble RDA for SAA for Low group for the 5-day repletion period while remaining in the GCRC inpatient unit.

Metabolic changes in response to SAA intake were detectable via ^1^H-NMR spectroscopy, and were investigated. Blood samples were drawn on each day after the 5 day equilibration period at 08:30. On the first and last days of the depletion and repletion periods, draws occurred 7 times after the morning meal at 08:30, 09:30, 10:30, 11:30, 12:30, 14:30, and 16:30. On these days, breakfast and lunch were combined into the morning meal. On the other days, samples were drawn only once a day after the morning meal at 8:30, and three meals and a snack were given. Since variation in redox increases in individuals over age 45, 6 female and 6 male subjects between the ages of 18 and 40 years old were recruited.

**Fig 4:**
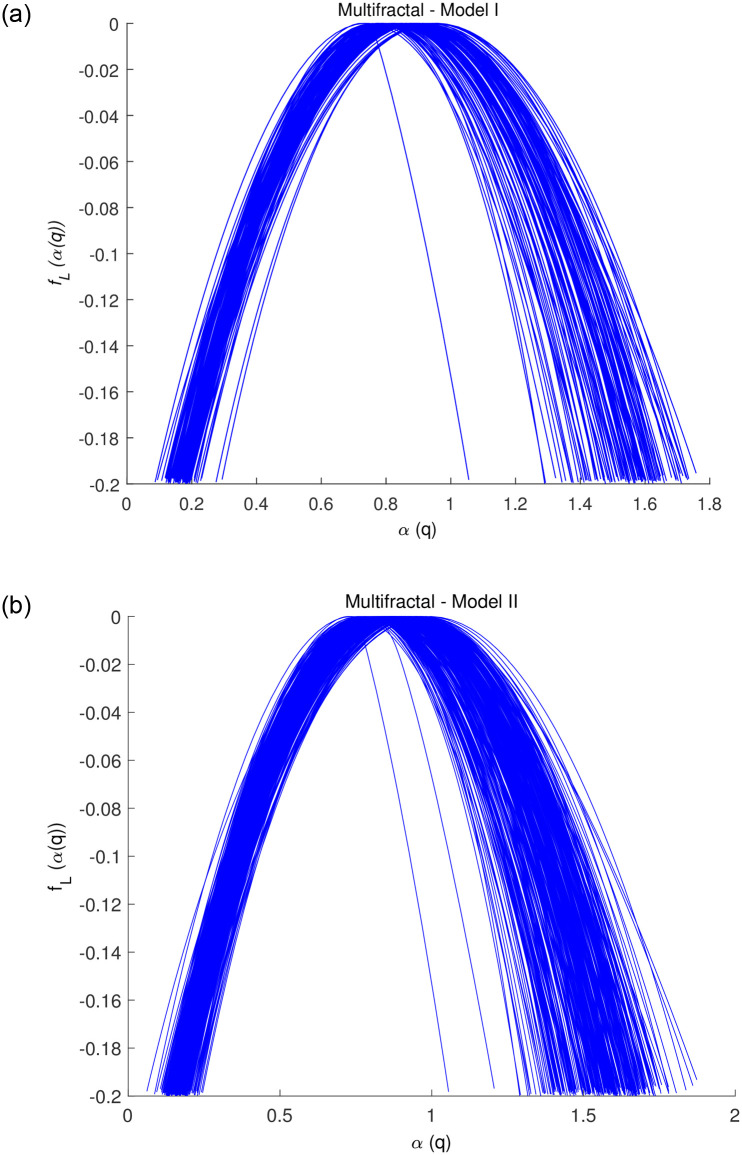
Multifractal spectra of ^1^H-NMR spectra Left: *Model-I* (Day Model, 120 spectra) and Right: *Model-II* (Hour Model, 336 spectra).

**Fig 5:**
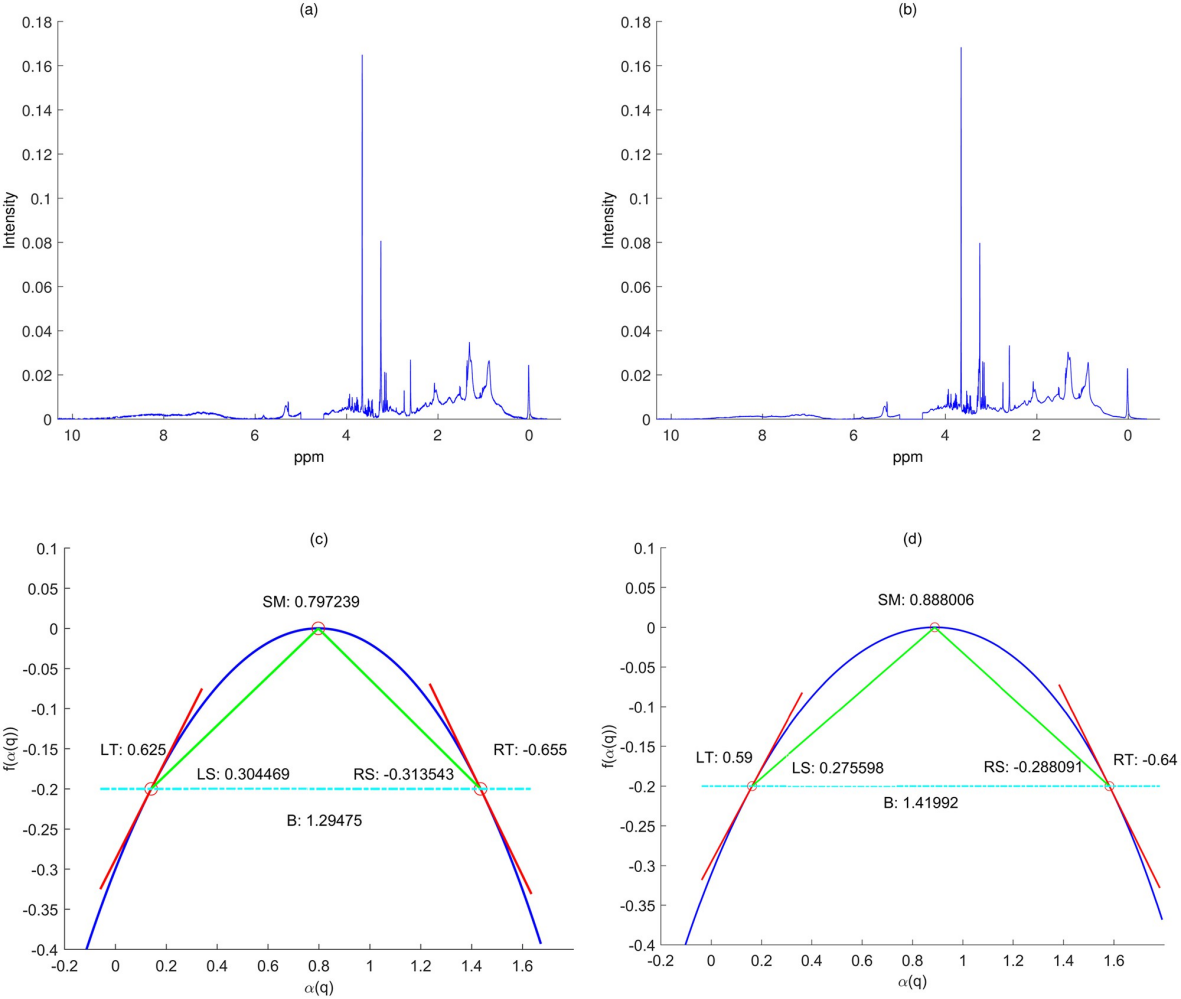
Illustration of utility of multifractal analysis: Panels (a) and (b) show ^1^H-NMR spectra with (a) high dose of SAA (repletion) and (b) with low dose of SAA (repletion), respectively. Panels (c) and (d) depict corresponding multifractal spectra resulting from signals in (a) and (b) respectively.

Fig 4 provides all multifractal spectra of ^1^H-NMR spectra used for *Model-I* (left panel, 120 ^1^H-NMR spectra) and *Model-II* (right panel, 336 ^1^H-NMR spectra) defined in the following section, and Fig 5 shows the two ^1^H-NMR spectra (high and low dose) of each repletion period and corresponding multifractal spectra. As shown in Fig 5, the Hurst exponent (*SM*) of ^1^H-NMR spectra of low dose is larger than that of high dose, while left slope (or left tangent) of high dose is larger than that of low dose.

### 3.2 Statistical models and evaluations

Depending on variation in a specific amount of time—either variation in data gathered over several days at the same time (Day Model), or variation in data gathered at multiple times in an individual day (Hour Model)—two statistical models for each geometric feature (*SM*, *LS* and *B*) in form of an unbalanced 3-way ANOVA model are appropriate. These models are appropriate because blood samples from each subject were drawn through depletion and repletion of either treble or decuple RDA for SAA intake. The outputs $y_{ijk}^{1l}$ and $y_{ijk}^{2l}$ are the values of each geometric feature of multifractal spectrum of ^1^H-NMR spectrum for each model, *l* = *SM*, *LS* and *B*. The statistical analysis is performed by MATLAB.

#### 3.2.1 Model-I. (Day Model)

In this model, the effect of day for each geometric feature (*SM*, *B* and *LS*) is of interest, and the corresponding 3-way ANOVA model without considering interaction effects for each geometric feature *l* can be defined as

$$\begin{array}{c} y_{ijkm}^{1l}=\mu^{1l}+\alpha_{i}^{1l}+\beta_{j}^{1l}+\gamma_{k}^{1l}+\epsilon_{m}^{1l} \end{array}$$ (8)

where *μ*^1*l*^ is the grand mean, $\alpha_{i}^{1l}$ is the effect of group *i* (High group *i* = 1, Low group *i* = 2), $\beta_{j}^{1l}$ is effect of depletion (*j* = 1) and repletion (*j* = 2), $\gamma_{k}^{1l}$ is the effect of *k*^*th*^ day (*k* = 1, …, 5) and $\epsilon_{m}^{1l}$ is assumed to be an independent normal variable with mean 0 and unknown variance $\sigma_{1l}^{2}$ for each geometric feature, *m* = 1, …, *n*_*ijk*_ and *n*_1*jk*_ = 4, *n*_2*jk*_ = 8 for all *j* and *k*.

Table 1 provides descriptive statistics for each geometric feature obtained from the multifractal analysis for each period and each group. As can be seen, means of *SM* and *B* of the Low group are much larger than those of the High group, while overall means of *LS* of the Low group are smaller than those of the High group. However, the differences of means of each feature within each group are relatively small. The *p*−values for each effect are tabulated in Table 3 by the analysis of 3-way ANOVA model for each geometric feature. The group effect is significant for all three features because different amounts of SAA were given to different groups in both equilibration and repletion periods. Contrastly, the difference between depletion and repletion of SAA for each group was not significant for all three features. This is because the blood samples considered in this model were drawn at 8:30 a.m. each day and the only snack was provided at 21:30, which means the effect of SAA disappeared by the time morning arrived and blood was drawn again. There was no significant variation over day in the morning for all three features. Thus Tables 1 and 3 imply that the amount of SAA taken in significantly affects ^1^H-NMR spectra which were evaluated by multifractal analysis, and that ^1^H-NMR spectra of the Low group have a significantly more regular pattern with larger variability when blood samples are drawn every morning over the depletion/repletion period. However, in the sense of regularity of ^1^H-NMR spectra, there is no significant difference between depletion and repletion of SAA within each group and over 5 days.

**Table 1:** Descriptive statistics of *Model-I* (Day Model).

	Statistics	High Group			Low Group		
		SM	LS	B	SM	LS	B
Depletion	Mean	0.8138	0.3052	1.3103	0.8785	0.2813	1.4327
	SE	0.0064	0.0025	0.0190	0.0061	0.0023	0.0133
Repletion	Mean	0.8060	0.3073	1.2883	0.8793	0.2800	1.4380
	SE	0.0077	0.0036	0.0250	0.0051	0.0017	0.0111
Overall	Mean	0.8099	0.3062	1.2993	0.8789	0.2807	1.4354
	SE	0.0050	0.0022	0.0156	0.0039	0.0014	0.0086

*Note*: SE = Standard Error, SM = Spectral Mode, LS = Left Slope, B = Broadness

#### 3.2.2 Model-II. (Hour Model)

Rather than using the effect of day as in Model I (Day Model), *Model-II* (Hour Model) assesses hourly variation as well as group effect and depletion/repletion effect. Similarly to previous model, the corresponding statistical model for each feature *l* can be expressed as

$$\begin{array}{c} y_{ijkm}^{2l}=\mu^{2l}+\alpha_{i}^{2l}+\beta_{j}^{2l}+\gamma_{k}^{2l}+\epsilon_{m}^{2l} \end{array}$$ (9)

where *μ*^2*l*^ is grand mean, $\alpha_{i}^{2l}$ is the effect of group *i* (High group *i* = 1, Low group *i* = 2), $\beta_{j}^{2l}$ is the effect of depletion (*j* = 1) and repletion (*j* = 2), $\gamma_{k}^{2l}$ is the effect of *k*^*th*^ hour slot (*k* = 1, …, 7), and $\epsilon_{m}^{2l}$ are assumed to be independent normal variables with mean 0 and unknown variance $\sigma_{2l}^{2}$ for each feature, *m* = 1, …, *n*_*ijk*_ and *n*_1*jk*_ = 8, *n*_2*jk*_ = 16 for all *j* and *k*.

Table 2 provides descriptive statistics of three geometric features of the Hour Model. Similarly to the Day Model, the mean values of *SM* and *B* of the Low group is larger than those of the High group, while the mean values of *LS* of the Low group are smaller. In addition, the differences of mean values within each group are relatively small but larger than those of the Day Model for all three features. Contrary to the Day Model, all three effects for *SM* feature in Table 3 are significant (*p*−values are <0.0001 for Group, 0.0205 for Depletion/Repletion, and 0.0268 for Hourly variation, respectively). That is, ^1^H-NMR spectra of the Low group has a significantly more regular pattern, the difference between depletion and repletion for the Hurst exponent (*SM*) is also significant, and hourly variation for the Hurst exponent is significant as well. Group effect for *LS* and *B* features is also significant. Thus, group effect is important for all three features, while the difference between depletion/repletion and hourly variation is significant for the Hurst exponent. This result can be well explained because subjects received the morning meal with decuple or treble RDA for SAA, which could affect metabolic changes in each day associated with sulfur amino acids.

**Table 2:** Descriptive statistics of *Model-II* (Hour Model).

	Statistics	High Group			Low Group		
		SM	LS	B	SM	LS	B
Depletion	Mean	0.8253	0.3014	1.3435	0.8906	0.2771	1.4547
	SE	0.0054	0.0023	0.0139	0.0038	0.0012	0.0083
Repletion	Mean	0.8085	0.3054	1.3157	0.8854	0.2778	1.4562
	SE	0.0044	0.0022	0.0147	0.0029	0.0011	0.0069
Overall	Mean	0.8169	0.3034	1.3296	0.8880	0.2774	1.4554
	SE	0.0036	0.0016	0.0101	0.0024	0.0008	0.0054

*Note*: SE = Standard Error, SM = Spectral Mode, LS = Left Slope, B = Broadness

**Table 3:** 3-Way ANOVA results.

Effects	Day Model			Hour Model		
	SM	LS	B	SM	LS	B
Group	**<0.0001**	**<0.0001**	**<0.0001**	**<0.0001**	**<0.0001**	**<0.0001**
Depletion/Repletion of SAA	0.7315	0.9584	0.8044	**0.0205**	0.2511	0.3959
Time (Day or Hour)	0.3387	0.4867	0.0869	**0.0268**	0.5947	**0.0118**

*Note*: The values in the table reflect the *p*−value.

### 3.3 Classification of ^1^H-NMR spectra

**Fig 6:**
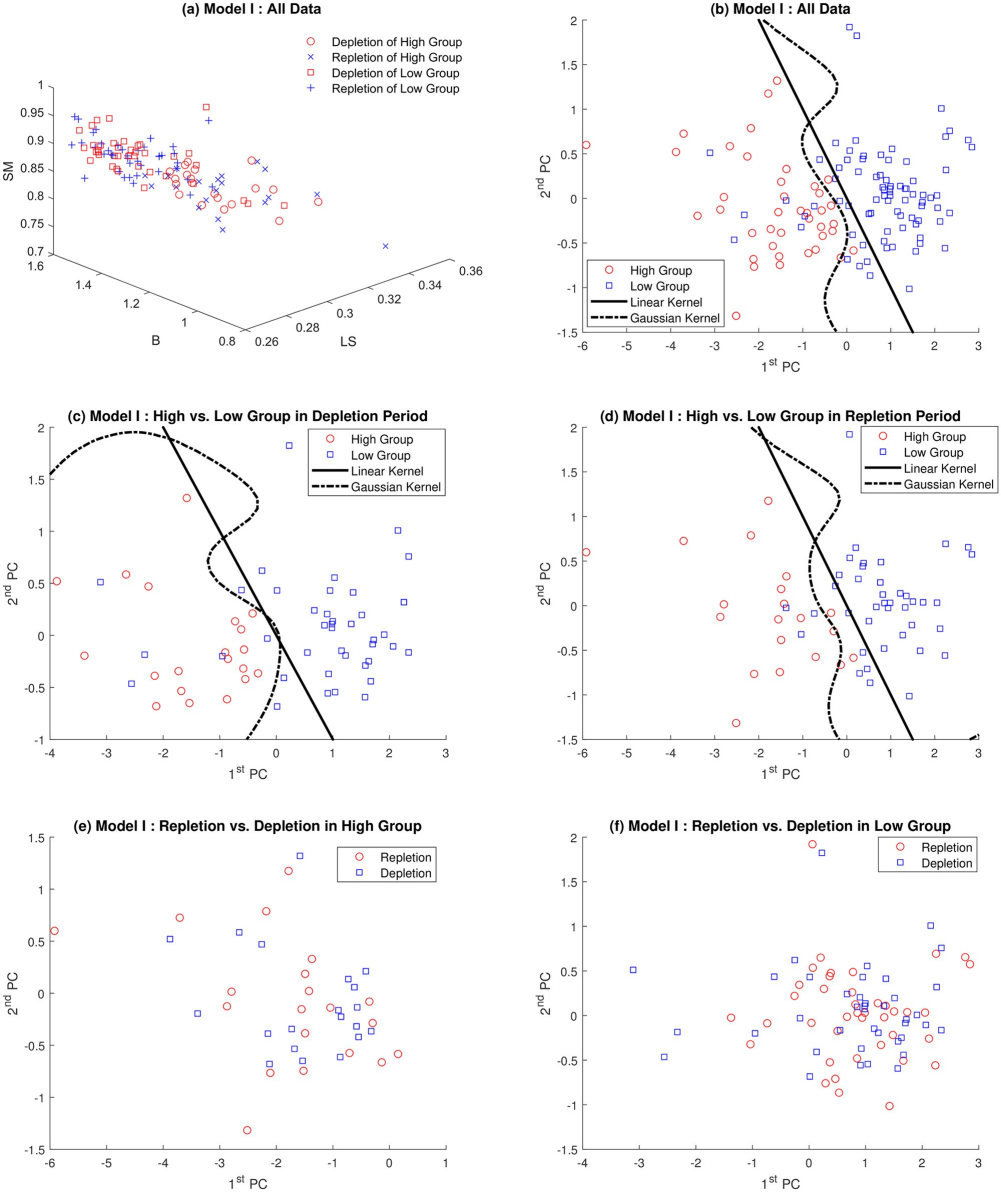
Classification of *Model-I* by PCA and SVM with linear and Gaussian kernels: Panel (a) represents all data together in 3 dimensions (*SM*, *B* and *LS*), (b) shows all of projected data onto plane spanned by first two principle components. Panels (c) and (d) reveal the classification of repletion and depletion data, respectively. Panels (e) and (f) show depletion/repletion data within High and Low group are indiscriminate by SVM, respectively.

**Fig 7:**
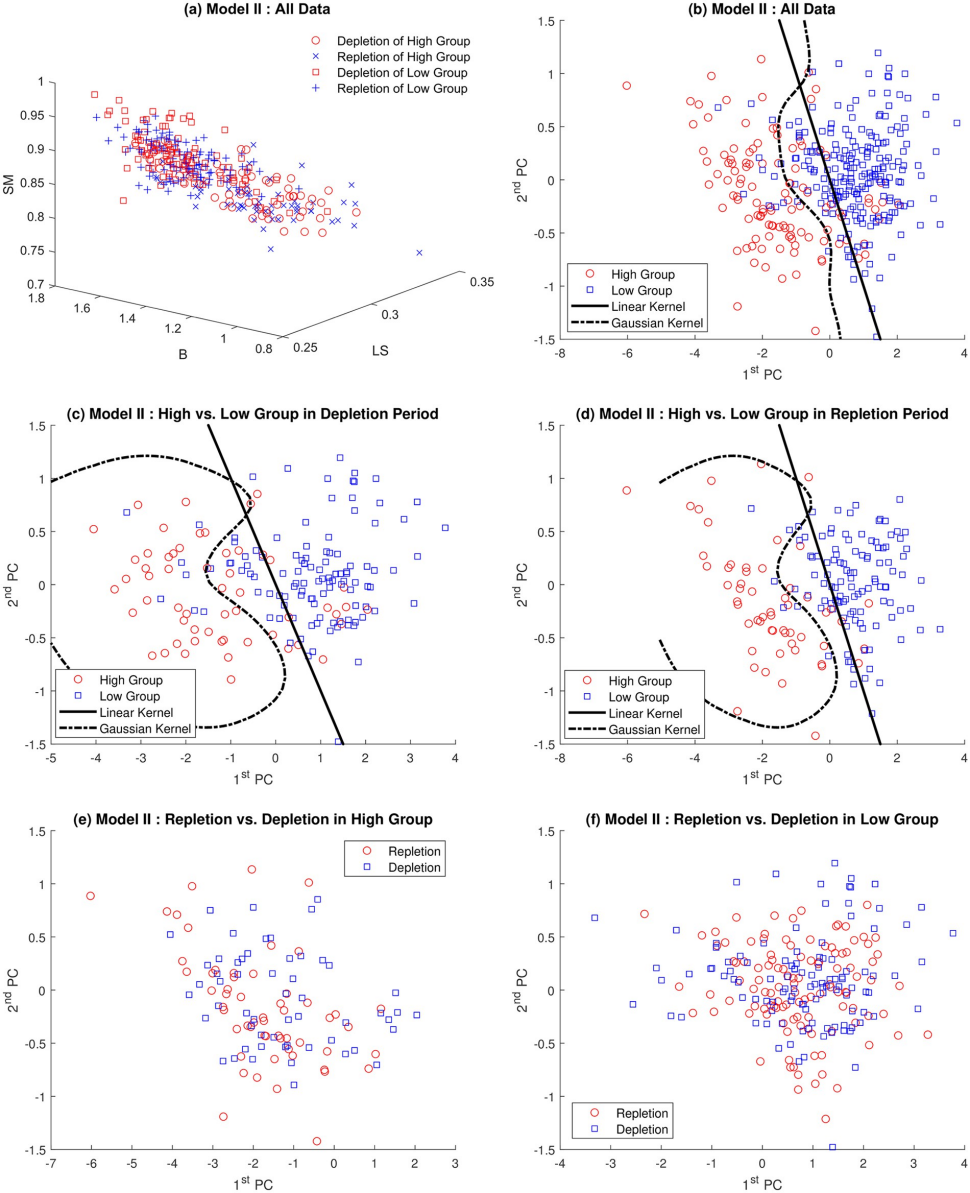
Classification of *Model-II* by PCA and SVM with linear and Gaussian kernels: Panel (a) represents all data together in 3 dimensions (*SM*, *B* and *LS*), (b) shows all of projected data onto plane spanned by first two principle components. Panels (c) and (d) reveal the classification of repletion and depletion data, respectively. Panels (e) and (f) show depletion/repletion data within High and Low group are indiscriminate by SVM, respectively.

As discussed before, multifractal analysis is focused on the distribution of the regularity indices *α*, rather than simply on the estimation of global and single index *α* [25]. In Section 3.2 the underlying contributions were assessed by wavelet based multifractal analysis of ^1^H-NMR spectra. Each ^1^H-NMR spectrum can be summarized in three geometric features defined in section 2.2 by Multifractal analysis. Panel (a) in Figs 6 and 7 represents all data in three dimensions (*SM*, *B* and *LS*). The High group data is located in the upper left corner, which implies that the High group is likely to be discriminated from the Low group.

Based on these observations, the Principal Component Analysis (PCA) methodology is employed to reduce dimension. As a result, 96.5% and 97.4% of variations of *Model I* and *Model-II* can be explained by first two principal components, respectively. Panel (b) shows all of projected data onto the plane spanned by first two principal components, which clearly shows plausibility of discrimination of spectra. After dimension reduction procedure, graphical discrimination by the Support Vector Machine (SVM) with linear and Gaussian kernels is conducted in panels (b)-(d). Panels (c) and (d) of Figs 6 and 7 demonstrate that two study groups in each depletion and repletion period are apparently discriminated. However, panels (e) and (f) shows that depletion and repletion data are indiscriminate within each study group.

The seven classifiers, SVM with linear and Gaussian kernels, Naive Bayes, Linear Discriminant Analysis, Quadratic Discriminant Analysis, *k*−Nearest Neighbor, and Decision Tree, are employed to classify all projected data into High and Low groups for each model. Table 4 summarizes the performance of these classifiers for comparison. The “All Data” shows the results when all data are used as the training set. The data seem highly discriminative as the area under ROC curve (AUC) values range from 0.947 to 0.983 for *Model-I* and from 0.909 to 0.985 for *Model-II*. In addition, the AUC values and classification errors by the 10-fold cross-validation (CV) are presented. One thousand replications are conducted to have a robust estimate of the AUC values and classification errors. The AUC values except for *k*−Nearest Neighbor by 10-fold CV are greater than 0.8 for both models, consistent with the 3-way ANOVA results.

**Table 4:** Discrimination by classifiers after the dimension reduction.

Classifiers	*Model-I*				*Model-II*			
	All Data		10-fold CV		All Data		10-fold CV	
	AUC	Error	AUC	Error	AUC	Error	AUC	Error
SVM with linear	0.947	0.108	0.864	0.120	0.911	0.131	0.828	0.133
SVM with Gaussian	0.952	0.075	0.913	0.091	0.914	0.128	0.820	0.141
Naive Bayes	0.947	0.142	0.814	0.153	0.909	0.128	0.834	0.133
Linear Discriminant Analysis	0.947	0.150	0.818	0.149	0.911	0.119	0.846	0.123
Quadratic Discriminant Analysis	0.948	0.142	0.819	0.147	0.912	0.131	0.838	0.132
*k*−Nearest Neighbor	0.983	0.083	0.727	0.212	0.980	0.095	0.763	0.190
Decision Tree	0.961	0.067	0.855	0.130	0.985	0.054	0.807	0.171

CV: Cross-Validation, Error: Classification Error, SVM: support-vector machine

## 4 Conclusions

This research aims to determine whether variation in sulfur amino acids (SAA) intake affects the metabolic process. Contrary to the traditional approach focusing on particular biomarkers, multifractal analysis of high-frequency and/or complex data has been supported by use in various areas [18, 22, 26]. In this study, two groups received food with different amounts of SAA during the equilibration and repletion period. Based upon three geometric features, *SM* (Hurst exponent), *LS*, and *B* of the multifractal spectrum, two statistical models for each feature were employed to assess underlying effects such as the group effect, depletion/repletion effect, and time effect.

In *Model-I*, the group effect is very significant for each feature, while variation over a day (the day effect) and depletion/repletion effect are not significant for all three features at all. However, in *Model-II* the group effect is significant for all features, while the depletion/repletion effect and hourly variation in each day are significant only for *SM*. This implies that the amount of SAA taken in is important and that SAA intake significantly affects the hourly variation of the metabolic process. Moreover, ^1^H-NMR spectra of the Low group have significantly more regular patterns with more variability than those of the High Group for both statistical models, which could be evidence that a high dose is recommended because the irregularity of spectra could be considered as the sign of good health. The relationship between the regularity of ^1^H-NMR spectra and the metabolic process could be important, but will be left for further research.

In addition, the seven classifiers followed by the PCA were employed to see if ^1^H-NMR spectra for both statistical models were discriminated. ^1^H-NMR spectra of the High and Low groups can be discriminatory for both models, while the spectra of depletion and repletion within each study group are indiscriminate for both statistical models. These results are consistent with those of 3-way ANOVA analysis except that of *SM* in *Model-II*, which is significant.

## Supporting information

S1 File (ZIP)
